# The Role of Topography in the Distribution and Intensity of Damage Caused by Deer in Polish Mountain Forests

**DOI:** 10.1371/journal.pone.0165967

**Published:** 2016-11-16

**Authors:** Radomir Bałazy, Mariusz Ciesielski, Krzysztof Stereńczak, Zbigniew Borowski

**Affiliations:** Forest Research Institute, Sękocin Stary, Braci Leśnej 3, 05–090, Raszyn, Poland; College of Agricultural Sciences, UNITED STATES

## Abstract

The increase in the deer population observed in recent decades has strongly impacted forest regeneration and the forest itself. The reduction in the quality of raw wood material, as a consequence of deer-mediated damage, constitutes a significant burden on forest owners. The basis for the commencement of preventive actions in this setting is the understanding of the populations and behaviors of deer in their natural environment. Although multiple studies have been carried out regarding this subject, only a few suggested topography as an important factor that may influence the distribution and intensity of deer-mediated damage. The detailed terrain models based on LiDAR data as well as the data on damage caused by deer from the State Forests database enabled thorough analyses of the distribution and intensity of damage in relation to land form in this study. These analyses were performed on three mountain regions in Poland: the Western Sudety Mountains, the Eastern Sudety Mountains, and the Beskidy Mountains. Even though these three regions are located several dozen to several hundred kilometers apart from each other, not all evaluated factors appeared common among them, and therefore, these regions have been analyzed separately. The obtained results indicated that the forest damage caused by deer increased with increasing altitude above 1000 m ASL. However, much larger areas of damage by deer were observed at elevations ranging from 401 to 1000 m ASL than at elevations below 400 m ASL. Moreover, the locations of damage (forest thickets and old stands) indicated that red deer is the species that exerts the strongest pressure on forest ecosystems. Our results show the importance of deer foraging behavior to the structure of the environment.

## Introduction

In the last three decades, increases in many deer populations have been noted on a global scale [1,2]. The results of many studies that were carried out in various locations throughout the world indicated that the high density of ungulates has strongly impacted many ecosystems, including forests [3,4,5,6,7]. Intensive pressure from deer modified not only forest regeneration, but also limited the diversity of the herbaceous vegetation, its layout and structure, soil characteristics and the deposition of mycorrhizal fungal spores. Finally, it has been stated that as a result of the change in plant cover, the diversity of insect, bird, and small mammalian species has decreased [2,8]. The high pressure of deer on a forest ecosystem is highly visible in many woodland ecosystems, which was documented in many previous studies [9–11,12, 13]. With respect to the structure of woodland ecosystems and deer feeding behavior, the increase in the level of damage caused by those animals was observed at early developmental stages of forest stands (young forest plantations and forest thickets) [14]. However, it seems that the pressure of deer on forest regeneration is not linear [15]. Aside from deer population density, the pressure on forest regeneration likely also depends on many external factors such as topographic features of the land, the level of anthropopressure (distance to the road), management activities and forest type [5,16, 17].

Despite the fact that elevation influences the ungulate’s feeding as the availability of alternative food supplies and weather conditions (wind and temperature) change, little is known about the impact of landscape features on the level of damage caused by these animals in the forest. For example, among studies related to damage caused by deer in the forests of Europe published in the years 1996–2012, only a few extensively discussed the issues of vertical distribution and topographic features of land [5]. Additionally, worldwide, few publications analyzed the role of elevation in ungulate foraging behavior and ungulate-mediated damage [18, 19, 20].

Of course, such analyses are not simple because elevation is a factor that impacts many different parameters that may have an important role in the pressure of deer on the forest ecosystem, e.g., climate, food resources, cover, physiological constraints and deer density (seasonal vertical migrations). Unfortunately, to date, no published work has analyzed the relationship between deer pressure, forest stand characteristics and elevation. Thus, in the present study, aiming to fill this data gap, we analyzed the vertical distribution of damage to forest stands caused by deer in three different study regions. The main questions of this study were as follows:

1. Is the level of damage similar between the three analyzed study regions?
2. On which forest stand is the strongest deer pressure?
3. How does elevation modify deer pressure on a forest ecosystem?
4. Does elevation influence the extent of deer-mediated damage?

## Materials and Methods

### Study Region

All studies were conducted at the lands belonging to the Polish State Forest Holding. All studies were funded by Polish State Forest Holding. Specific permissions were not required for these locations because only remote sensing data and data base were used.

The study region included three different parts, which are located in the western (51°10'0''N, 15°12'37''E) and southern portions of Poland (49°21'56''N, 19°27'37''E). All study regions are characterized by the dominance of forest spruce monocultures, which formed as a consequence of purposeful and faulty human operation at the turn of 19^th^ and 20^th^ centuries.

**Fig 1:**
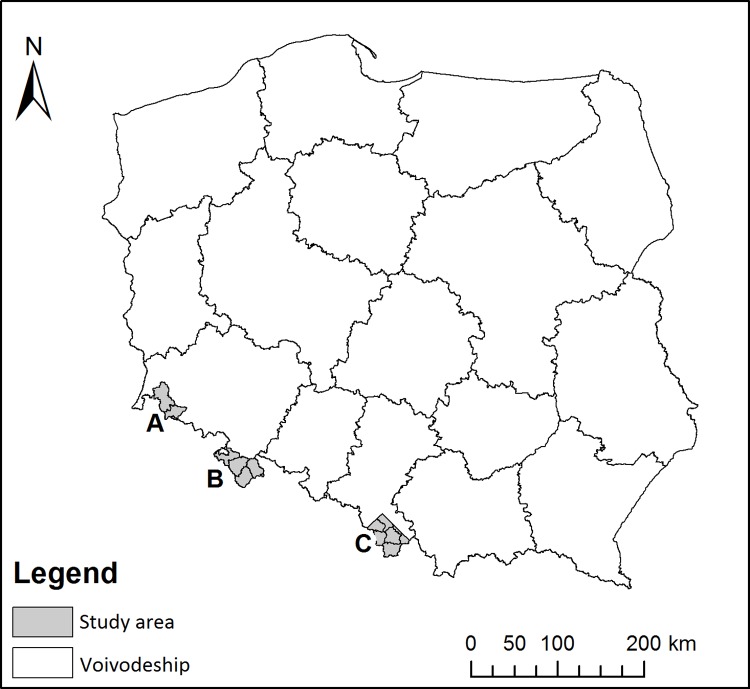
Location of the three study regions in the territorial divisions of Poland.

The study region covers twelve mountain forest districts located in the Sudety and Beskidy Mountains, and due to their geographical location and distinct forest management history, they have been divided into three areas (Fig 1):

- Area “A”–the Western Sudety Mountains: Forest Districts Szklarska Poręba and Świeradów;
- Area “B”–the Eastern Sudety Mountains: Forest Districts Bystrzyca Kłodzka, Zdroje, Międzylesie, and Lądek Zdrój;
- Area “C”–the Western Beskidy Mountains: Forest Districts Bielsko, Jeleśnia, Ujsoły, Ustroń, Wisła, and Węgierska Górka.

**Fig 2:**
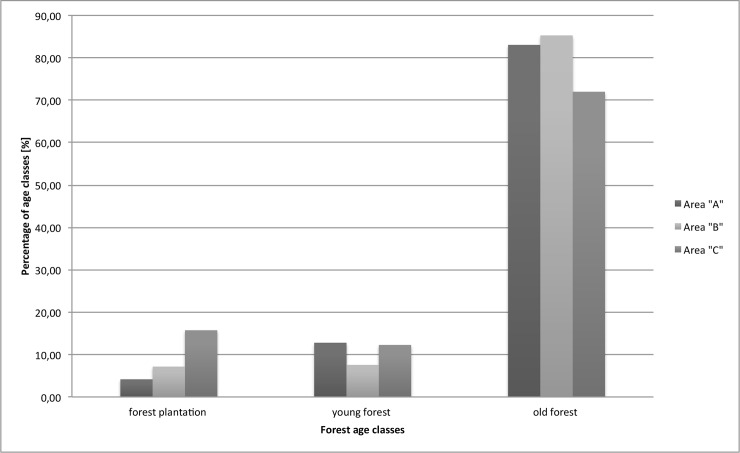
Distribution of the forest age classes at three study regions.

The Western Sudety Mountain region (Area “A”) is an area that was affected by large-scale deforestation that caused air pollution in the 1980s (over 15,000 hectares). As a result, there is a distorted age structure and a relatively large proportion of trees of the same age class in this forest stand (Fig 2). The Western Beskidy Mountain region (Area “C”) is currently affected by the decline of spruce stands, caused by various factors (natural decline, pollution, and monoculture of trees), which resulted in an increased proportion of trees in younger age classes–mainly in plantations. Spruce stands, the least distorted among all tree stands, remain unchanged in the Eastern Sudety Mountains (Area “B”). Due to the transformation of original forest stands into spruce monocultures and their increasing age, this region is facing the possible threat of massive deforestation as a result of future disturbances.

Even more surprisingly, the three areas did not differ in the distribution of urban areas according to elevation; therefore, in every region, a similar area of damage relative to the total forest area may be observed. The three regions did not significantly differ in the distribution or the size of forest complexes.

According to the geographical divide of Poland into sections [21], the study regions are located within the sections of Pogórze Zachodniosudeckie and Western Sudety (Area “A”), Eastern Sudety (Area “B”), and Western Beskidy (Area “C”). Western Sudety and Eastern Sudety may be treated similarly as far as plant cover is concerned. In those areas, the mid-storey vegetation was divided as follows: the foothills zone up to 500 m AMSL, the lower forest zone at 500–1000 m AMSL, the upper forest zone at 1000–1250 m AMSL, the dwarf pine zone at 1250–1450 m AMSL, and the alpine zone above 1450 m AMSL. In the foothills zone, there are lowland and highland types of forest stands. In the upper forest zone, coniferous forest stands are dominant in the mountains, but in the lower forest zone, there are only mixed coniferous forest stands in the mountains. The main forest species in those areas is the Norway spruce *Picea abies*. Other major species include the beech *Fagus sylvatica*, the oak *Quercus robur*, the fir *Abies alba*, the pine *Pinus sylvestris*, and the black alder *Alnus glutinosa*. Western Beskidy, located in the Carpathian Mountains, is characterized by a different division of story vegetation. The foothills zone is up to 600–700 m AMSL, the lower forest zone is 600–1150 m AMSL, and the upper forest zone is 1150–1400 m AMSL. The dominating forest species in this area is also the Norway spruce.

### Animal Density Estimation

The study regions also differed in terms of deer densities. Information about the deer species composition and their densities was collected from official game inventories in spring 2013 based on the same methodology—deer drive counts [7] by hunting associations in the study area.

The red and roe deer populations in Western Sudety (70 thousand ha) differed from those in the other two regions. The largest population of red deer was in Eastern Sudety (containing the greatest number of forest complexes, 10.75 deer per km^2^), which also had the largest culling. Whereas the Western Sudety regions had similar numbers of red deer, the Western Beskidy region had the highest roe deer density (Table 1). However, the combined red and roe deer densities were similar between Eastern Sudety and Western Beskidy. The great asymmetry in deer number may have resulted from differences in the forest area, which is nearly twice as large in Eastern Sudety than in Western Sudety. It may also be an artifact of variable densities throughout the established range.

**Table 1:** Density of red and roe deer in the three study regions: Areas A, B and C per 1 km^2^ of forest area.

Study Area	Red deer	Roe deer	Red and roe deer
		
Western Sudety(A)	387 = 0,553 for km^2^	311 = 0,444 km^2^	0,997 /km^2^
Eastern Sudety (B)	1268 = 1,075 for km^2^	969 = 0,821 km^2^	1,896/ km^2^
Beskidy (C)	562 = 0,472 km^2^	1732 = 1,455 km^2^	1,927/km^2^

### Spatial Data

The research was based on data acquired from the State Forests Information System regarding the extent of damage caused by deer. These data were obtained from field inventories performed by forest services from April to May 2013. Damage events included in the database related to main three categories: browsing, gnawing or breaking tree trunk and bark stripping. The area of damage caused by deer registered in forest stands was classified into three groups (up to 20%, 21–40%, and above 40% of all trees); however, only the two groups corresponding to most damage (> 21%) were registered according to Instruction of Forest Protection [22]. In addition, damage was reported independently for each of the three developmental phases of tree stands: forest plantation, forest thicket, and old forest stand. It should be emphasized that current and historical damage events were collectively evaluated on a cumulative basis. The data used in this study were acquired in the first quarter of 2013 (S1 Table).

Section surfaces, species composition, and other elements of the survey description, such as age, were acquired from the Forest Digital Map as a shape file. The estimated extent of damage was calculated according to the adopted criterion of the elevation above sea level; this was possible using a digital terrain model (DTM) with a ground sampling distance of 0.5 m. The DTM was interpolated from airborne laser scanning data acquired in 2012. According to the performed analyses, the accuracy in the elevation using the developed Digital Terrain Model (DTM) was approximately 20 cm (S1 Table).

### Data Processing

The data were processed in three phases.

- In the first phase, damage caused by mammals was analyzed. The intensity of damage caused by Carvidae species was divided into classes: 21–40% and >40%. The percentage represents the amount of damage caused on each plot. Because the classes are relatively wide, the authors decided to reduce the area of damage by adding variables to selected classes: 30% for the class 21–40%, and 70% for the class > 40%. In the data from the central database of the Polish State Forests (SILP) regarding the extent of damage by animals, for every district in which damage events were documented, data regarding the developmental phase of the forest stands were available. For the districts in which damage events were not registered, missing data were supplemented regarding the developmental phase of the forest stands, adopting the criterion of the age of the dominant species in a given district. All districts in which the age of the dominant species was ≤10 years, were assigned to the growing forest plantation group. Forest districts aged between 10 and 20 years were assigned to the young forest group, and the remaining districts were assigned to the old-forest group.
- The second phase of the study was the acquisition of information regarding the terrain morphology for individual districts. For this purpose, a tool was used, i.e., “*zonal statistics as table”* (Spatial Analyst Tools, Zonal) implemented in ArcMap 10.3 software, which enabled the calculation of average levels of elevation above sea level. To generalize the detailed data and prepare the data for statistical analyses, five classes of elevation above sea level were distinguished for each district: 0–400, 401–600, 601–800, 801–1000, and 1001–1200 m above sea level. Every district was then allocated to the relevant class of elevation.
- In the last step, statistical analyses were carried out using Statistica 10.0 software (http://www.statsoft.com/Products/STATISTICA-Features/Version-10) with a significance threshold of P = 0.05. From these analyses, final results were categorized according to the extent of damages for the three specified study regions (Areas A, B, and C) and were divided into three phases of a forest stand development (growing forest plantation (up to 10 years old), forest thicket (from 10 to 20 years old), and older forest stand (above 20 years)), as well as divided into the classes of elevation defined above. Due to the linear relationship between the forest area damaged by deer and the proportion of land covered by forest at a given elevations (y = 5,3378 + 0,4868*x; r = 0,5370; p < 0,00011), the forest area damaged by deer (ha) was weighted by the total forest area (ha). This proportion was a dependent variable, whereas study region (Areas A, B, and C), phase of the forest stand (plantation, thicket, and old forest), elevation (<400, 401–600, 601–800, 801–1000 and >1000 m) and compass directions (North, South, West, and East) were independent variables. A one-way ANOVA was used for evaluation of differences among variables, followed by Bonferroni and Games–Howell post hoc tests for homogenous and heterogeneous variances, respectively. General linear models (GLMs) were mainly applied to the log-transformed data to better approximate a normal distribution.

## Results

**Fig 3:**
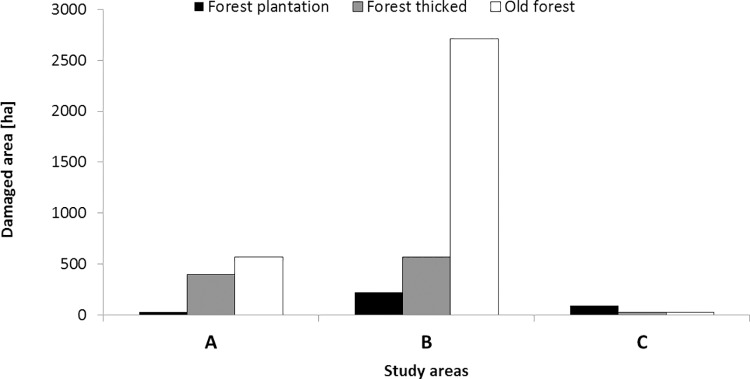
The levels of forest damages of different age classes (forest plantation, forest thicket and old forest stand) caused by deer in the three study regions.

The three investigated regions differed in terms of total area of damage caused by deer in forests. The greatest damage events were observed in the Eastern Sudety Mountains (study area B). Considerably less damage was noted in Western Sudety (study area A), and marginal damage occurred in the Beskidy Mountains (study area C) (Fig 3). In the Eastern and Western Sudety Mountains, the greatest area of damage caused by deer was observed in older forests (over 20 years old) and in forest thickets, and the least damage was observed in forest plantations (Fig 3). By contrast, in the Beskidy Mountains, where damage by deer was minimal, the greatest area of damage was observed in forest plantations.

In addition, to better characterize the three investigated study regions with respect to the pressure exerted by deer on the forested land, the ratios of damaged forest area observed in to the total forest area were calculated for tree stands of each developmental phase (Table 2).

**Table 2:** The ratio of observed damaged forest area to total forest area for tree stands of each developmental phase in three analyzed study areas.

Study areas	Forest stand	Forest area [ha]	Damaged forest area [ha]	Ratio of forest damages [%]
**Western Sudety(A)**	Forest plantation	945,2	56,819	6,0
	Forest thicket	2597,93	483,921	18,6
	Old forest	20408,54	667,083	3,3
	total	**23951,67**	**1207,823**	**5,0**
**Eastern Sudety (B)**	Forest plantation	3411,98	406,433	11,9
	Forest thicket	3534,04	726,258	20,6
	Old forest	41351,03	3268,192	7,9
	total	**48297,05**	**4400,883**	**9,1**
**Beskidy (C)**	Forest plantation	6223,73	130,434	2,1
	Forest thicket	4866,71	34,635	0,7
	Old forest	30824,26	31,553	0,1
	total	**41914,7**	**196,622**	**0,5**

As is apparent from Table 2, the three investigated study regions differed in terms of the total forest area, and the extent of damage by animals depended on the total forest area within the given region (F_(1,7)_ = 6,267, p<0,0408).

**Fig 4:**
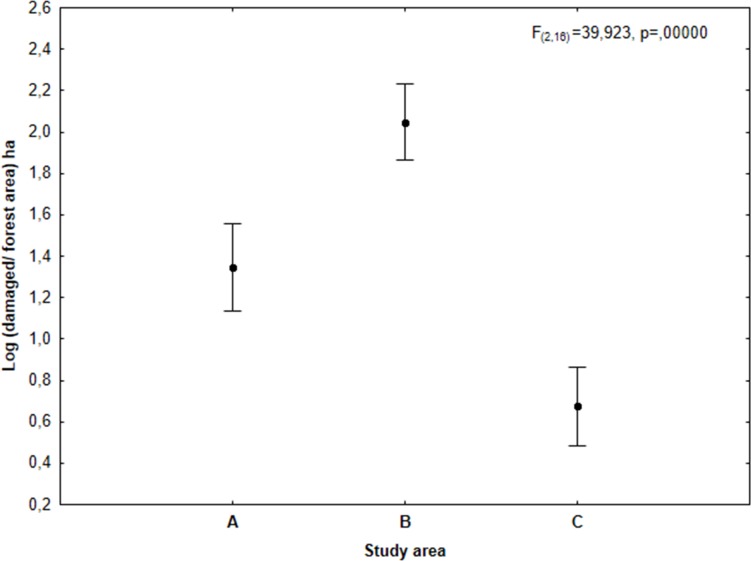
The size of log-transformed area of forest damage (weighted by total forest area) caused by deer in the three different study regions: Western Sudety (A), Eastern Sudety (B) and Western Beskidy (C).

**Fig 5:**
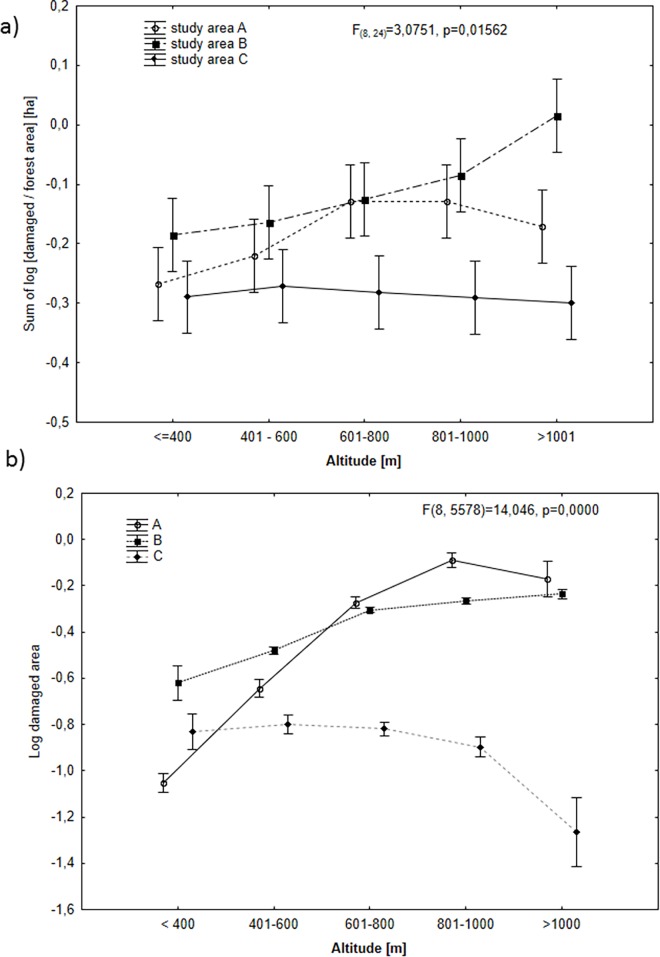
Relationship between elevation and: (a) Log-transformed total area of forest damages caused by deer; (b) Log-transformed mean (± SD) size of individual forest areas damaged by deer in three study regions (Western Sudety (A), Eastern Sudety (B) and Beskidy (C)).

The analysis of damage severity calculated for each forest area indicated that the greatest pressure on forests was exerted by deer in Eastern Sudety, smaller one in Western Sudety and the least pressure was exerted in the Beskidy Mountains (GLM, F_(2,8)_ = 31,035, p = 0,0002) (Fig 4). No difference in damage severity was observed with respect to the developmental phase of the tree stands (forest plantation, forest thicket, and old forest stand over 20 years old) in each of the study areas F_(4, 24)_ = 2,466, p = 0,0722). However, in Eastern Sudety (study area B) the severity of damage was found to depend on elevation (F_(8, 24)_ = 3,0751, p = 0,0156) (Fig 5A). Significantly more damages were recorded on areas at elevations above 1000 m ASL, while areas situated below 400 m ASL (Boniferroni exact test p = 0,008) and between 401 to 600 m ASL (Boniferroni exact test p = 0,0290) were characterized by lower level of damage.

To determine the spatial distribution of pressure from animals, differences in the size of individual areas damaged by deer were analyzed. Contrary to the previous parameter, the average area, not the total area, damaged by deer was compared in this analysis. Given that this parameter differed significantly among the various study regions (ANOVA, F_(6, 5584)_ = 54,196, p<0,0001), statistical analysis was performed separately for every terrain.

**Fig 6:**
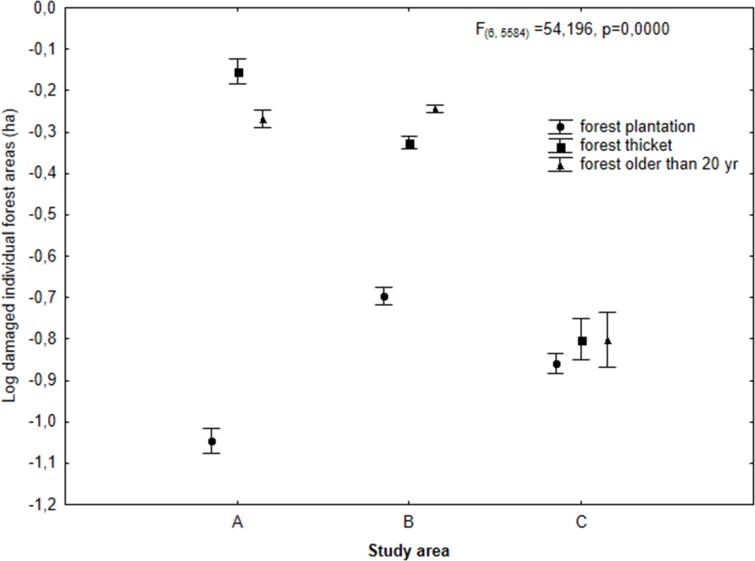
Log-transformed mean (±SD) size of individual forest areas damaged by deer in relation to total forest area and stratified according to age class: forest plantation, forest thicket and old forest stand (ANOVA, F_(6, 5584)_ = 54,196, p<0,0001).

In Western Sudety, the greatest average areas of tree stands damaged by deer were observed in forest thickets; slightly less damage was observed in old forest stands (over 20 years old), and the least damage was found in forest plantations (F_(2,1048)_ = 240,870, p = <0,0001). In Eastern Sudety, the greatest damaged areas were noted in old forest stands and forest thickets, and the least damaged forests were plantations (F_(2,4005)_ = 233,740, p = <0,0001). However, in the Beskidy Mountains, no significant difference was noted in the average damaged areas between the developmental phases of the tree stands (ANOVA, F_(2,521)_ = 1,3063, p = 0,2717) (Fig 6).

In the Western Sudety region, the average area damaged by deer depended on the elevation (ANOVA, F_(4, 1046) =_ 96,298, p<0,0001). The greatest damaged areas were observed at elevations above 600 m ASL Considerably less damage was observed at elevations between 401 and 600 m ASL, and the least damage was noted at the lowest elevations (< 400 m ASL) (Fig 5B). In the Eastern Sudety region, a very similar pattern was observed: the least damaged areas were noted at elevations up to 600 m ASL, and the greatest damaged areas were observed above this elevation (Fig 5B; GLM: F_(4, 4003)_ = 32,378, p<0,0001). In the third region, Area C, no differences in the area damaged by deer was observed between elevations (Fig 5B. F_(4, 529) =_ 2,422, p>0,05).

**Fig 7:**
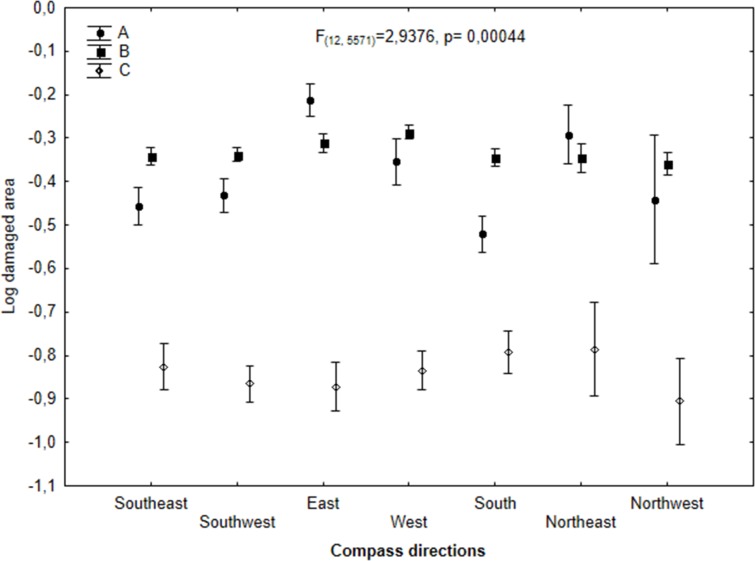
Log-transformed sizes (mean ± SD) of individual forest areas damaged by deer in relation to compass directions for each studied region (Western Sudety (A), Eastern Sudety (B) and Beskidy (C)).

In mountain regions weather conditions (wind,solar radiation) are related with compass directions and might have the impact on deer foraging behavior. That is why in this study we have analyzed the impact of compass directionson the monitored areas with damaged forest stands. This parameter significantly affected the size of areas damaged by deer solely in Eastern direction of Western Sudety region, (Fig 7, GLM F_(6, 1044)_ = 6,269, p<0,0001). In contrast, it this parameter did not impact damage areas with respect to elevation or developmental phase of the tree stands (p>0,05). In the other two study regions, the compass directions did not impact the level of damage caused by deer (Fig 7, Area B: GLM F_(6, 4)_ = 1,617, p = 0,1381; Area C: GLM, F_(6, 527)_ = 0,4043, p = 0,8763).

## Discussion

As shown, the three investigated study regions differed not only in the population and species composition of inhabiting deer but also in the size of the damage caused by these mammals in forests. The greatest area of damage was observed in Eastern Sudety; a significantly lower level of damage was noted in Western Sudety, and the least amount of damage was observed in the Beskidy Mountains. In addition, the greatest damage was observed in forest thickets and old forest stands (over 20 years old). These results indicate red deer as the species that was responsible for this damage [23]. Interestingly, very little damage was observed in forest plantations, which was different than lowlands, where damage events on forest plantations caused by foraging roe deer were dominant. A probable cause this phenomenon may be that during a period when damage events occur in forests (i.e., late fall, winter, and early spring), there is thick snow cover in the mountains, which constitutes an effective barrier against deer foraging and thus protects seedlings. This hypothesis seems not to apply to the Beskidy Mountains, where damage events in plantations were comparable to damage events in older tree stands. In addition, in the Beskidy Mountains, the proportion of forest area in this age class, i.e., forest plantations, among the total forest area was the highest.

Given that the total damage area is directly related to the size of the analyzed forest area, damage caused by deer was presented as the ratio of damaged surface to the total forest area within the forest district. In this sense, the greatest pressure is exerted by deer on the forest ecosystem in Eastern Sudety, with significantly less pressure on forests in Western Sudety and the least pressure on forests in the Beskidy Mountains. These results correspond well with the observed deer density. According to official hunting statistics, Eastern Sudety had the highest density of red deer out of the three analyzed regions. There is a question why in Beskidy Mountains, where red deer population density is on similar level to Western Sudety and roe deer density is much higher, there is the lowest level of forest damages caused by deer. One possible explanation of this phenomenon may be a difference in the environmental structure and the foraging base available for deer between regions. Therefore, if the Western Sudety Mountains offer lower foraging base for deer than Beskidy Mountains, the animals were more willing to consume an alternative food in the form of trees. An alternative explanation for this phenomenon may be the discrepancy between the actual red and roe deer population density in each study region and the population density estimated by hunters (based on official hunting statistics).

By analyzing damage in certain developmental phases of tree stands, it was possible to determine the particular species that caused the damage. In the study regions, two deer species are found, red and roe deer, but only red deer are able damage to trees by stripping bark in forest thickets and old forest stands (over 20 years old) [23]. Therefore, it may be assumed that the areas of greatest damage observed in Eastern and Western Sudety in forest thickets and forest old stands (over 20 years old) were related to red deer.

As mentioned above, the total area of damage and the average area of damage showed similar trends, as far as the location of damage is concerned. This is indicated by the fact that in the winter season, deer preferred forest thickets and old forest stands, where the animals foraged and stayed in one place for a long time., in turn, the total area of damage was highest in these types of tree stands. Alternatively, Western Beskidy contained the smallest total area of damage and the smallest average area of individual damage events. This may be related to the current state of the local tree stands, which were less attractive feeding grounds because they were more extensively degraded and thus exhibited greater anthropopressure.

Previous research demonstrated that the elevation above sea level has an impact on the area of damage, as observed by Pépin [24] and Beck [19]. An interaction between damage and elevation above sea level was noted in one region only—Eastern Sudety. The smallest proportion of damaged area was observed at the lowest elevations up to 600 m ASL, and the proportion of damaged area increased with elevation above 1000 m ASL. The opposite relationship was observed in the studies by Nopp-Mayr [25] and Beck [19], who justified the presence of greater damage at lower elevations by the fact that during the winter season, animals move to lower elevations where it is warmer and there are more supportive conditions. An increase in the proportional area of damage with increasing elevation was observed by Takeuchi [20], and this finding may be explained by a considerably smaller home range within which those animals remain in the winter season. This seems reasonable because in the mountains, the availability of alternative food sources decreases in the winter season with increasing elevation. Deer compensated for reduced food availability by stripping and eating tree bark.

It needs to be emphasized that bark does not provide optimal nutrition to deer, and it is consumed as so-called hunger feed, which animals use when basic feed sources are not available. Tree bark may constitute up to 10–15% of a deer’s nutrition [23]. The Western Sudety and Western Beskidy study regions differed completely from the Eastern Sudety in the vertical distribution of damage caused by deer, most likely due to the mild pressure of deer on forest ecosystems or the observed decline in spruce stands in that region. In these regions, no differences were observed in the vertical distribution of damage caused by deer.

Because the force and speed of wind and the level of sunshine may have a strong impact on the foraging strategies of deer, the distribution of damage was analyzed with regard to compass directions (linked with weather conditions ie. Wind, solar radiation). However, the considerable influence of this factor was exclusively observed in Western Sudety, where the greatest damaged area was in the east direction. Therefore, an obvious question arises: what is the reason for the difference in the significance of compass directions in relation to foraging volume among the investigated study regions? Unfortunately, because such dependencies were not investigated here, it may only be assumed which factors might have influenced this observation. Because greater deposition of precipitation from fog is noted in Western Sudety than in the other regions [26], Western Sudety is the region in which hoarfrost occurs most often. In particular, hoarfrost is most commonly observed on the windward side, i.e., on the north-west, west, and south-west sections. Intensified foraging by deer may occur in the sections that are less exposed to hoarfrost.

However, no relation was observed between the level of damage and the compass directions, the elevation or the developmental phase of the tree stands. This result indicates that wind has likely not played any significant role in determining the extent of damage. How do we explain the observed lack of a relationship between the compass directions and the degree of damage? The reason for this might be the fact that deer foraged mainly in forest thickets and old forest stands, ensuring good thermal protection. An additional explanation is the fact that we did not have relevant weather data connected with compass directions allowing us to establish this kind of relationship In conclusion, the three analyzed mountain regions differ considerably, as pressure exerted by deer on the forest ecosystem is concerned. It seems that distinct environmental conditions, mostly the difference in the availability of feed during the winter season, constituted a key factor in the observed damage, which may explain the observed differences in damage between regions. The vertical differentiation in the proportional area damaged by deer indicates that the pressure of deer increases with increasing elevation. This relationship is likely a result of the decrease in the availability of winter feed. The location of damage in forest thickets and old forest stands indicates that the species that exerts the strongest pressure on forest ecosystems is red deer. The results of the research presented here show the importance of environmental structure, particularly the availability of a winter foraging base, to the interaction between deer and the forest ecosystem and to the pressure that animals exert on this ecosystem.

## Supporting Information

S1 Table Table including ID, age class and topographic data of all analysed forest parcels, extent of the reduced damages. (XLSX)
